# Field Responsive Swelling of Poly(Methacrylic Acid) Hydrogel—Isothermal Kinetic Analysis

**DOI:** 10.3390/polym17192602

**Published:** 2025-09-26

**Authors:** Jelena D. Jovanovic, Vesna V. Panic, Nebojsa N. Begovic, Borivoj K. Adnadjevic

**Affiliations:** 1 Institute for General and Physical Chemistry, Studentski Trg 12-16/V, 11158 Belgrade, Serbia; nbegovic@iofh.bg.ac.rs; 2 Innovation Center of Faculty of Technology and Metallurgy, University of Belgrade, 4 Karnegijeva Street, 11000 Belgrade, Serbia; vpanic@tmf.bg.ac.rs; 3 Faculty of Physical Chemistry, University of Belgrade, Studentski Trg 12-16, 11158 Belgrade, Serbia; adnadjevic@mts.rs

**Keywords:** hydrogel, isothermal kinetic, poly(methacrylic acid), microwave, ultrasonic, simultaneous cooling, swelling

## Abstract

Externally governed hydrogel swelling is a highly convenient yet inherently challenging process, as it requires both responsive materials and appropriately tuned external stimuli. In this work, for the first time, the influence of simultaneous action of external physical fields—ultrasound (US) and microwave heating (MW), combined with cooling—on the isothermal swelling kinetics of poly(methacrylic acid) (PMAA) hydrogel was investigated and compared with swelling under conventional thermal heating (TH) under isothermal conditions. Swelling kinetics were monitored over a temperature range of 248–318 K, under simultaneous cooling with either US, MW, or TH. The well-established Peppas model was used to determine swelling kinetics parameters, revealing a significant acceleration in the swelling process under MW (up to 48.8 times at 313 K), as well as different water penetrating mechanisms (non-Fickian diffusion) compared to TH and US (Super-case II). Additionally, it was demonstrated that the swelling conversion curves could be mathematically described using a “shrinking boundary surfaces” model. Isothermal swelling constants and the corresponding kinetic parameters (activation energy *E_a_* and pre-exponential factor ln *A*) were calculated. The results confirmed that external physical fields significantly influence the thermal activation and swelling behavior of PMAA xerogels, offering insight into field-responsive transport processes in hydrogel networks.

## 1. Introduction

Smart hydrogels are an extraordinary group of three-dimensional, cross-linked polymeric structures that, in addition to the ability to absorb a significant amount of water or other biological fluids, without dissolving or losing their structural integrity (swelling), also possess many unique properties [1,2]. They include softness, porosity, and biocompatibility, and resemble living tissues better than any other synthetic biomaterial. However, these advanced materials are considered smart and special primarily because they can undergo significant and reversible changes in their physical or chemical properties in response to external stimuli. Most often, they exhibit drastic swelling or de-swelling in response to environmental changes, often described as volume collapse or phase transition—a behavior that is more easily controlled in synthetic polymer-based hydrogels due to their well-defined composition and reproducible synthesis [2,3].

These stimuli include variations in pH, temperature, light, electric or magnetic fields, as well as the presence of specific ions, molecules, or enzymes. The responsiveness of these hydrogels to such environmental stimuli makes them particularly valuable in the field of biomedicine, sensors, agriculture, packaging, etc. Therefore, hydrogels are excellent potential materials for many applications such as drug delivery, cell culture and encapsulation, wound dressing, scaffolds for tissue engineering, soft actuators, environmental protection, and green energy materials. [4,5,6,7].

For instance, pH-sensitive hydrogels have found significant applications in targeted drug delivery, where they can release medication selectively in the slightly acidic environment of tumor tissues, in the acidic environment of the stomach, or in neutral to alkaline environments, like the intestines and blood. Similarly, temperature-responsive hydrogels can undergo transitions around body temperature, making them ideal for use in injectable drug systems, wound dressings, and tissue engineering scaffolds [8]. Light-responsive hydrogels offer precise, non-invasive control over drug release by utilizing specific wavelengths to trigger structural changes within the polymer network. This photo-modulation enables spatiotemporal regulation of therapeutic delivery, enhancing efficacy while minimizing systemic side effects [9].

Additionally, hydrogels that respond to electric or magnetic fields with their tunable, reversible behavior enable real-time adaptation in biomedical applications, such as targeted drug delivery, tissue engineering, and soft robotic actuators, thereby enhancing therapeutic precision and patient safety. Beyond healthcare, these hydrogels are also used for controlled water release in agriculture, the detection of pollutants, the design of advanced sensor systems, etc. [10,11]. However, most of the reported literature data deals with applicative properties, such as the effect of the use of a magnetic field on drug release from hydrogel [12,13], and not on the swelling process itself under the different external fields.

Although smart hydrogels sensitive to the above-mentioned stimuli have found their application and are of great interest to researchers and manufacturers, science continues to seek both new materials sensitive to easily accessible, effective, and non-invasive external stimuli, as well as new external stimuli [14].

Among the non-conventional, energy-efficient sources of energy that are most frequently used nowadays are microwave and ultrasonic fields. Although operating on different principles, both of them considerably accelerate chemical reactions and physico-chemical processes and lead to higher product yields, often with improved properties and better selectivity [15,16].

US and MW fields can non-invasively penetrate biological tissues, offering a distinctive advantage in externally controlling hydrogel behavior—particularly in modulating swelling and enabling precise, on-demand drug release within drug delivery systems (DDS) [17].

Jovanovic and Adnadjevic have investigated the kinetics of nicotinamide release from poly(acrylic-co-methacrylic acid) xerogel under the conditions of simultaneous MW and cooling [18]. The investigated release process has been modeled as a first-order chemical reaction. Release rate constants (*k*) were determined experimentally at various temperatures and found to range from 8.4 × 10^−3^ s^−1^ to 15.7 × 10^−3^ s^−1^. Using these temperature-dependent k values, an Arrhenius analysis was performed under the conditions of simultaneous MW and cooling, confirming that the Arrhenius equation applies even in that environment. From this analysis, the activation energy (*E_a_*) has been calculated as 25.6 kJ/mol, as well as the pre-exponential factor (ln *A* = 5.21 s^−1^). It has also been demonstrated that the higher rate constants observed under MW compared to conventional TH were not due to system overheating or localized “hot spots” [18,19].

Noguchi and Takaomi [20] have found reversible changes in the cellulosic hydrogel under the US field. They have reported the deformation of the gel structure in cellulosic hydrogels, caused by the 43 kHz ultrasound exposure, monitored as a significantly reduced G′ modulus of the hydrogel. The declined G′ returned to the original value after the action of the US field, indicating the reversibility of the process. Jiang et al. [21] have investigated the effects of the US field with different powers and frequencies on the release of mimosa from the cellulose-based hydrogel matrix. Changes in hydrogel structures have been found after the US field action. Monitoring the viscoelasticity of the hydrogel has shown that it became more rigid after the US exposure, while FT-IR analysis has revealed that the structure of mimosa–water and mimosa–cellulose was somewhat changed.

It has been shown that hydrogels can undergo changes in their applicative properties, mainly mechanical and structural, when exposed to the US or MW fields of sufficiently high energy [18,19,20,21]. But what if the applied energy level is adjusted so as not to change the structure?

Among the most striking and widely studied properties of hydrogels, swelling of hydrogels in water, aqueous solutions, and biological fluids is one of their main properties, which is decisive in terms of their inner structure, influences the majority of other properties, and defines their possibilities and potential applications. The swelling kinetics of various hydrogels have been extensively investigated in terms of the effects of pH value of swelling mediums, temperature, ionic strength, etc. [22,23,24,25,26,27].

To the best of our knowledge, no research has investigated the effect of the US and MW fields on hydrogel swelling. The effect of the US field on water (solution) is reflected in US energy dissipation in solution, resulting in cavitation, which includes the nucleation, growth, and collapse of bubbles in solution. When a swelling hydrogel network is exposed to a US field, cavitation-induced solvodynamic shear will significantly affect the dynamics of polymer chain segments within hydrogel networks. When collapsing, a bubble pulls nearby polymer segments toward its cavity, and the resulting shear forces stretch out the polymer backbone. At sufficiently high ultrasonic energies, this elongation can lead to chain scission; however, by adjusting the US power to avoid polymer degradation, the same mechanism can be harnessed to modulate hydrogel swelling. Ebrahimi et al. have reported that the level of US irradiation influence on polymer depends on various experimental factors, including temperature, solvent, and sonication intensity, because the generated vibrational wave energy, shear stresses at the cavitation interface, and localized high pressure and temperature can contribute to the change in polymer structure [28]. These are also the major factors that can influence the swelling behavior of hydrogels under the US field. Similar effects could be expected to influence hydrogels’ swelling under the microwave field. 

Poly(methacrylic acid) is a well-known anionic polyelectrolyte that has been used in hydrogel formulations of unique properties: extensive swelling, softness, good cell proliferation, adsorptive and mucoadhesive properties, non-toxicity (FDA approval), biocompatibility, the capability to respond to environmental pH changes, etc., which make them favorable for use in many fields [29]. The swelling of PMAA-based hydrogels has been investigated in terms of the effects of composition, crosslinker type and concentration, pH value of swelling medium, temperature, ionic strength, etc., on equilibrium swelling degree and swelling kinetics [28,29,30,31]. Most of the data have been reported in so-called conventional environments, i.e., in the thermal field, under isothermal conditions, and described by the well-known Peppas kinetics model.

To the best of our knowledge, this study is the first to investigate hydrogel swelling under US and MW fields and to show that the swelling of PMAA hydrogel could be influenced by US and MW fields. The significance of the examination of swelling under MW and US lies in the possibility of applying hydrogels that can be combined with US or MW fields, such as in drug delivery or environmental protection. On the other hand, due to the enormous exposure of the planet Earth to different types of radiation, especially MW (radars, mobile phones, etc.), and bearing in mind the structural similarity of hydrogels with living tissues, the investigation of the effects of MW and US fields on hydrogels can be of exceptional importance. This research aims to elucidate the effects of MW and US fields on hydrogel swelling behavior, potentially expanding the application scope of stimuli-responsive hydrogels, but also providing insights into the interaction of such fields, which we are significantly exposed to, with tissue-mimicking materials.

## 2. Materials and Methods

***Materials:*** Methacrylic acid (99.5%) was purchased from Merck KGaA, Darmstadt, Germany. N,N′-methylenebisacrylamide (MBA) (p.a.) and sodium hydroxide (p.a.) were obtained from Aldrich Chemical Co., Milwaukee, WI, USA. The initiator, 2,20-Azobis-[2-(2-imidazolin-2-yl)propane] Dihydrochloride (VA-044) (99.8%) was supplied by Wako Pure Chemical Industries, Ltd. Poly (methacrylic acid) (PMAA) hydrogel was synthesized according to the procedure described in [29].

***Isothermal swelling experiments*** **.**

The isothermal kinetic swelling experiments were undertaken under the thermal, ultrasonic, and microwave fields at a temperature range of T = 293–318 K ± (0.1–1) K, keeping the temperature constant. The well-known tea-bag method was applied [23] to determine the kinetic swelling. In general, the swelling experiments are conducted as follows. At the beginning, dried hydrogel samples (xerogel) in the form of disks were weighed (*m*_0_) (average weight 0.05 g) and immersed entirely in excess distilled water at constant temperature, and left to swell. At predetermined time intervals, samples were taken out from the water, wiped with soft paper to remove excess superficial water, and weighed (*m_t_*). The measurements were performed until equilibrium was reached. The equilibrium swelling degree (SD*_eq_*) was calculated as follows:

SD_*eq*_ = (*m*_*eq*_ − *m*_0_)/*m*_0_ (1)

And normalized swelling degree was calculated as follows:

*α* = SD/SD*_eq_* (2)

All of the measurements were performed using three samples, and the mean values are given.

***Swelling under the TH*** **.**

In the case of conventional TH, the isothermal conditions of the experiment were achieved so that the swelling was carried out in the thermostat with precision control of temperature, and continuous temperature measurement was carried out in the medium (in this case, distilled water) where the swelling took place.

***Swelling under the MW field*** **.**

The study of the swelling kinetics of PMAA hydrogel under the conditions of MW field with simultaneous cooling was performed on a modified microwave device—Discovery—produced by CEM corporation, Matthews, NC, USA, at a working frequency of 2.45 GHz. The detailed description of the working principle of the modified microwave device has been provided in the work of B. Koturevic et al. [16], and the scheme of the modified microwave device used in this investigation has been shown in the work of J. Jovanovic [18].

***Swelling under the US field*** **.**

The study of the swelling kinetics of PMAA hydrogel under the action of US with simultaneous cooling was performed in a thermostatically controlled ultrasonic reactor (Model VC 750 made by Sonics and Materials Inc., Newtown, CT, USA) with 20 kHz working frequency and 0–750 W power; amplitude setting was displayed in % on a scale of 10–100. The ultrasonic reactor consists of a titanium alloy ultrasonic probe with a 13 mm diameter tip. The schematic presentation of an ultrasonic reactor with a simultaneous cooling system has been presented in the work of the same research group [32].

***Swelling kinetics models*** **.**

The isothermal kinetic curves of swelling were fitted with the most frequently used model—Peppas’ kinetic model [23]—as well as with a relatively new boundary phase shrinkage model.

Peppas’ swelling kinetics model is presented by the power law equation:

(3) $$\alpha =k\times \;t^{n}$$

where *α* = SD/SD*_eq_* denotes the fraction of water absorbed by the hydrogel at time *t*. The constant *k* captures the hydrogel’s structural and geometric properties, defining the swelling rate. The swelling exponent n reveals the mechanism by which water penetrates the polymer network (whether dominated by diffusion, polymer relaxation, or both).

For a planar hydrogel, the meaning of *n* is as follows:

- *n* ≤ 0.5—Fickian (diffusion-controlled) swelling, where water diffusion into the polymer is much faster than the polymer chains’ rearrangement.
- 0.5 < *n* < 1.0—Anomalous (non-Fickian) swelling, where both diffusion and polymer relaxation contribute.
- *n* ≥ 1.0 Case II transport, controlled by relaxation or swelling-front propagation within the polymer [23].

Originally introduced for the analysis of drug release from polymeric matrices, the Peppas model has since been extensively employed to describe the swelling kinetics of hydrophilic polymer networks. Its applicability extends across various classes of hydrogels, including physically and chemically crosslinked systems, owing to its ability to empirically capture deviations from ideal Fickian behavior in the early stages of swelling. In particular, the model has proven valuable in evaluating the swelling dynamics of pH-sensitive and polyelectrolyte hydrogels such as those based on methacrylic and acrylic acids.

Despite its empirical nature, the Peppas model remains a cornerstone in hydrogel swelling analysis, particularly when the full analytical solution of Fick’s second law is impractical. However, it is generally valid only up to about 60% of the total swelling time, after which deviations can occur due to relaxation-dominated or non-linear effects.

The so-called model of “contracting area”, which is characteristic of phase-boundary controlled reactions with a bidimensional shape, provides an alternative theoretical framework for describing the swelling kinetics of polymeric hydrogels, particularly in planar geometries. This model assumes that the rate-limiting step in the swelling process is the movement of a distinct swelling front, with the area of the unswollen core decreasing over time. The name ‘contracting area’ arises from the geometric interpretation in two-dimensional systems, where the area of the dry polymer shrinks progressively as water diffuses inward. The time-dependent swelling behavior can be described by the following expressions:

(4) $$kt=1-{\left(1-\alpha \right)}^{\frac{1}{2}}$$

where *α* = SD/SD*_eq_* represents the fraction of water absorbed by the hydrogel at time *t*, while *k* is the rate constant.

This model thus offers valuable insights into systems where swelling behavior is dominated by interfacial dynamics rather than homogeneous diffusion, so classical diffusion models fail to capture the observed kinetics. This approach has been shown to successfully model swelling in polyelectrolyte hydrogels containing methacrylic and acrylic acid [22].

## 3. Results and Discussion

The swelling kinetics of PMAA-based hydrogel were systematically evaluated across different experimental conditions involving external fields.

**Figure 1:**
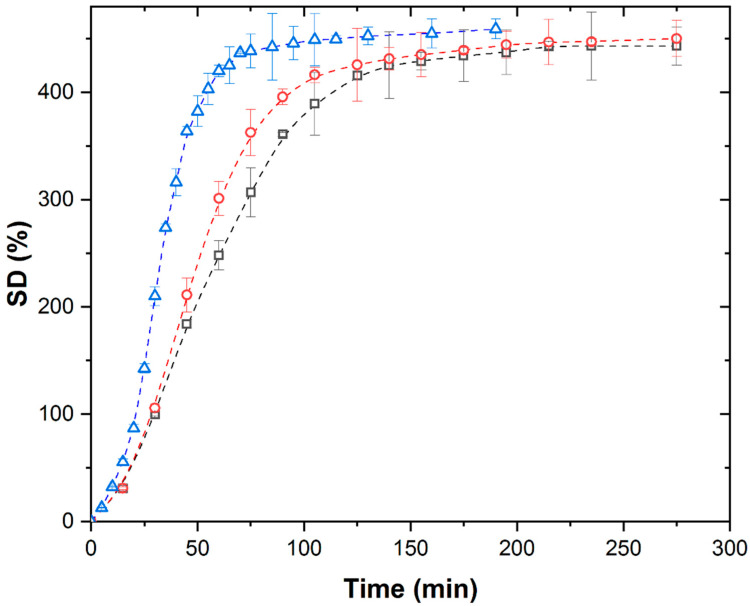
Isothermal swelling kinetic curves of PMAA xerogel in distilled water under TH at 298 K (∀), 308 K (-), and 313 K (8).

Figure 1 shows the isothermal kinetic curves of swelling of PMAA xerogel in distilled water under a conventional field of thermal heating at different temperatures.

The swelling kinetic curves at all investigated temperatures exhibit a characteristic shape, the so-called S-shape, consisting of four distinct stages: convex, linear, concave, and plateau regions. The increase in temperature results in a slight increase in the values of equilibrium swelling degree from 443 g/g to 459 g/g, while shortening the duration of each characteristic stage and reducing the time required to reach equilibrium swelling degree.

**Figure 2:**
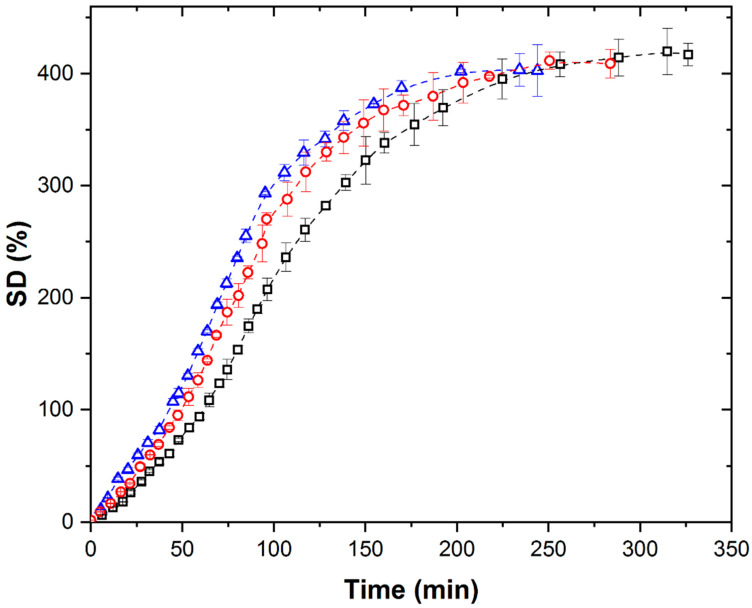
Isothermal swelling kinetic curves of PMAA xerogel under US with simultaneous cooling at (∀) 298 K, (-) 308 K, and (8) 313 K.

The isothermal kinetic curves of PMAA xerogel swelling in distilled water were recorded at different temperatures under an ultrasonic field (US) at 40 kHz with simultaneous cooling, as shown in Figure 2.

Under US conditions, the swelling curves retain the S-shape at all temperatures, but the SD*_eq_* shows minimal dependence on temperature, as well as the duration of the characteristic regions and the time to reach equilibrium. The SD*_eq_* values reached under the US field were 7.0, 10.5, and 12.3%, which were lower than the corresponding SD*_eq_* values under TH. This suggests that ultrasound exerts an influence on swelling kinetics that is distinct from temperature effects alone. Although we do not have direct experimental evidence, our results suggest that cavitation is the key factor responsible for the observed temperature-independent behavior of SD*_eq_* under ultrasound. Since the only difference between the thermally (TH) and ultrasonically (US) treated systems is the presence of cavitation, we attribute the suppression of temperature dependence to cavitation effects. These include localized high pressures, microstreaming, and rapid energy deposition, which appear to dominate over the effects of bulk temperature alone.

It is most likely that US, due to cavitation effects, induces a nonequilibrium distribution of energy between the internal (intra-molecular groups of oscillating atoms) and external (oscillations of network segmental—chain, pendant groups) degrees of freedom. This happens because the energy transfer in the system is faster than the establishment of thermodynamic (thermal) equilibrium and leads to the establishment of a non-equilibrium distribution of energy according to the degrees of freedom of the system. Namely, when hydrogels swell in water under ultrasound, cavitation (the formation and violent collapse of microscopic bubbles) can create highly localized zones of elevated temperature and pressure. These short-lived conditions introduce energy into the system too rapidly for it to be immediately and evenly distributed. As a result, some of the energy is first absorbed by fast internal motions within polymer chains (such as bond vibrations or side group rotations), while larger-scale processes like polymer relaxation and water diffusion follow more slowly. This leads to a temporary non-equilibrium between intra- and inter-molecular energy pathways.

This nonequilibrium energy distribution stimulates internal forces that drive energy exchange through shear and volume deformations, which significantly affect polymer chain segment dynamics within the hydrogel network. Because of this, nonequilibrium distributions have an affinity for establishing an equilibrium distribution of energies by degrees of freedom through the mutual exchange of energies between the degrees of freedom.

Unfortunately, at the current level, it is not possible to provide direct experimental evidence of the described energy distribution, but some indirect evidence, such as enhanced diffusion, formation of radicals, and structural changes in ultrasound-treated polymers, supports the idea that cavitation alters energy distribution during swelling.

When collapsing, cavitation bubbles attract nearby polymer segments toward their cavity, and the resulting shear forces can mechanically stretch out the polymer backbone. Besides that, more space for water molecules to penetrate can be created, facilitating water penetration and triggering structural relaxation via rearrangement of intermolecular and supramolecular structures in the hydrogel due to the flow of intermolecular structures (segments) into new positions (structural relaxation). Most likely, this structural relaxation presumably decreases the cross-linking density of the network and increases the distance between the macromolecular chains (ξ). Consequently, the value of SD*_eq_* is independent of the swelling temperature.

**Figure 3:**
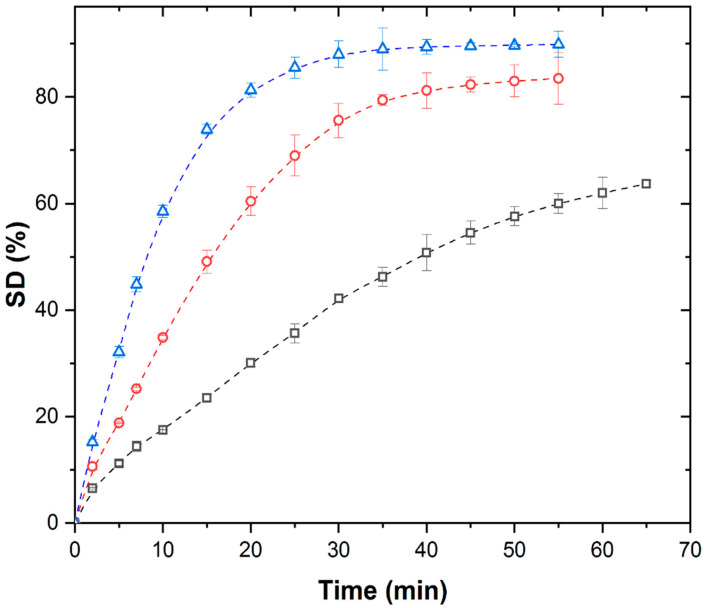
Isothermal swelling kinetic curves of PMAA xerogel under MW field with simultaneous cooling at (∀) 298 K, (-) 308 K, and (8) 313 K.

Figure 3 shows the isothermal kinetic curves of PMAA xerogel swelling at different temperatures under the conditions of simultaneous action of the MW field and system cooling.

Under MW conditions, swelling kinetic curves of PMAA xerogel at all the investigated temperatures exhibit a diminished sigmoidal shape that disappears at lower temperatures.

Contrary to TH and US, increasing temperature significantly enhances SD*_eq_* and shortens swelling times. However, SD*_eq_* values under MW remain substantially lower (by 81–85.6%) than under TH or US at comparable temperatures (Table 1).

These effects likely stem from MW-induced structural changes in the hydrogel network and increased mobility of water molecules due to selective absorption of MW energy by Na^+^-carboxyl groups and water molecules [5].

These effects are specific to the MW field compared to the TH and US fields, and most probably are the consequence of structural changes in the xerogel/hydrogel (due to the absorption of MW energy) and a significant increase in the mobility of water molecules, which is also caused by the absorption of MW energy. Structural changes in the xerogel/hydrogel triggered by the action of the MW field are most likely, as in the case of the US field, a consequence of the rapid, selective absorption of MW energy by the ions of Na^+^-carboxyl groups and water molecules existing in the PMAA xerogel/hydrogel network [5].

Structural relaxations induced by exposure to MW energy led to changes in the structure of PMAA hydrogel: a decrease in network cross-linking densities, and an increase in the value of the distance of the macromolecular chains (ξ) and in the rate of network relaxation, resulting in an increase in SD*_eq_*. To gain a more comprehensive understanding of the swelling behavior of hydrogels—a process inherently complex and further influenced by external stimuli such as US or MW fields—it would be desirable to go beyond conventional gravimetric swelling measurements. However, while in situ techniques such as X-ray diffraction (XRD), scanning electron microscopy (SEM), Fourier-transform infrared spectroscopy (FTIR), or dynamic mechanical analysis (DMA) would provide valuable insights, their application during swelling under US or MW conditions is currently not feasible. Nevertheless, the proposed swelling kinetics experiments, combined with appropriate mathematical modeling, can yield critical parameters, such as the mechanisms of water penetration, swelling rate, and activation energy. These data can serve as a solid foundation for evaluating and discussing the influence of external fields on hydrogel swelling behavior. We have an ongoing investigation using solid state nuclear magnetic resonance (SSNMR) methods to achieve a deeper, and hopefully novel, insight into structure–property changes and interactions during the swelling process, and we strongly believe in its potential.

In swelling experiments under US and MW fields, the absence of localized overheating in MW and US fields was ensured by using simultaneous cooling. While additional evidence, such as real-time thermal imaging, thermocouple data, or calorimetric validation, would strengthen the argument for non-thermal effects of US and MW fields, we currently lack access to such specialized equipment. Moreover, no available system can monitor all relevant parameters during swelling under both fields in real-time. However, several observations support the assumption that localized heating or ‘hot spots’ did not play a significant role [18,33]. Additional rheological and reswelling tests under TH at 298 K, performed on samples swollen previously under thermal, US, and MW fields, showed comparable results, indicating no structural degradation of the hydrogels. SEM images further confirmed the preservation of hydrogel morphology reported in [34]. Additionally, previous theoretical estimates of localized temperatures in MW-assisted nicotinamide release studies [18] have suggested unrealistically high temperatures, well above the polymer’s degradation threshold, implying that if such “hot spots” had occurred, they would have led to observable degradation, which was not the case. Since no such degradation was observed here and considering its potential impact on swelling behavior, we conclude that localized heating was most probably not a significant factor in this study.

In scientific literature, it is most often assumed that the swelling process is commonly controlled by the following factors: (a) the rate of diffusion of water molecules into the spatial cavities, (b) the migration of water molecules into the dynamically formed free spaces of the hydrogel/xerogel, and (c) the relaxation processes in the hydrogel, described by Peppas’ theoretical model [23].

**Figure 4:**
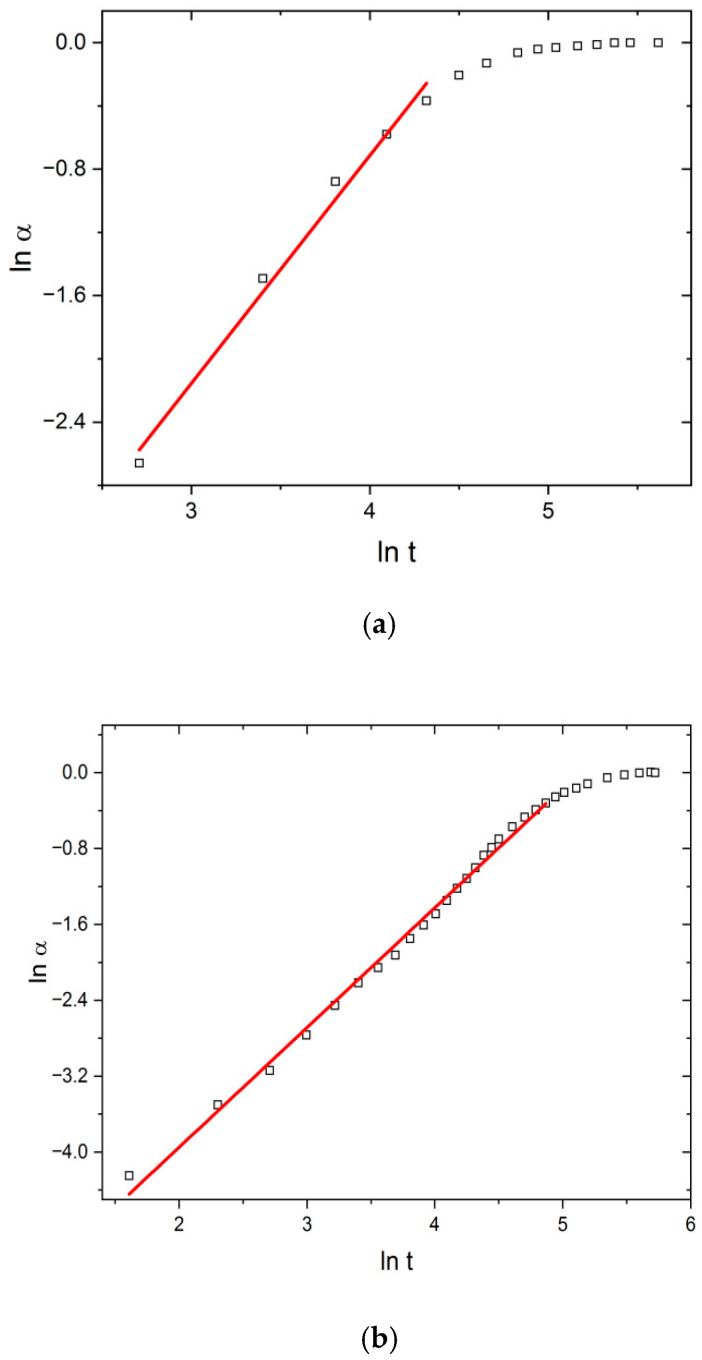

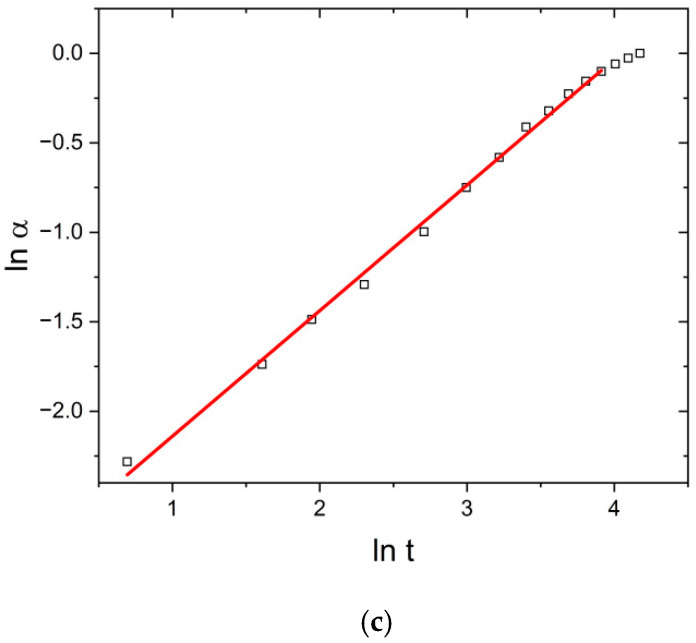
Peppas plots (ln *α* vs. ln *t*) under (**a**) TH, (**b**) US, and (**c**) MW fields at 298 K.

Regarding that, to determine the effect of US and MW fields with simultaneous cooling, the swelling data were transformed into Peppas’ conversion curves: ln *α* vs. ln *t*, shown in Figure 4, which represents TH, US, and MW fields at 298 K.

Linear behavior in the α range 0.05–0.85 confirms the applicability of the Peppas model to describe swelling kinetics and enables the calculation of parameters *k* and *n* (Table 1).

Table 1 shows the influence of the temperature, US, and MW fields on the values of the parameters of the Peppas model. Under the effect of temperature change alone (T increase from 298 to 313 K), *k*_p_ increased from 1.44 to 2.03, indicating the acceleration of the swelling process. At the same time, relaxation was a controlling step in the swelling process (Super case II transport), as the parameter n stayed above the value of 1, i.e., it slightly increased from 1.44 to 1.56.

Under the effect of the US field, Peppas’ kinetic parameters range from *k*_p_ = 1.54 (T = 298 K) to *k*_p_ = 3.84 (T = 313 K), while *n* ranges from 1.15 (T = 313 K) to 1.26 (T = 298 K). The increase in the value of *k*_p_ and the decrease in the value of the parameter n in relation to the values under the thermal field alone indicate that structural changes occur in the network during the operation of the US field. It is most likely that these changes are caused by cavitation effects, due to which the cross-linking density decreases relatively little. As a result, the SD*_eq_* value changes, and the swelling speed also changes.

In contrast, the values of the parameters *k*_p_ and *n* under the MW field significantly differ in relation to their corresponding ones under the TH and US fields. The values of *k*_p_ range from 58.3 to 99.2 (for T = 318 K), while *n* ranges from 0.70 (T = 298 K) to 0.80 (T = 313). The determined values of the diffusion parameter “*n*” under MW field conditions indicate non-Fickian transport, i.e., the dominant influence of the rate of diffusion of water molecules into the xerogel/hydrogel and the transport of water through the dynamically formed spaces of the xerogel/hydrogel on the swelling rate. This directly confirms the induced structural changes in the PMAA hydrogel network caused by the selective non-equilibrium absorption of MW energy by the PMAA network.

Although the Peppas model effectively describes up to ~85% of swelling, it cannot fully capture the entire process. Motivated by previous work [22,23,24], a novel kinetic model incorporating boundary phase shrinkage [35,36] was applied to better fit the full swelling curve.

**Figure 5:**
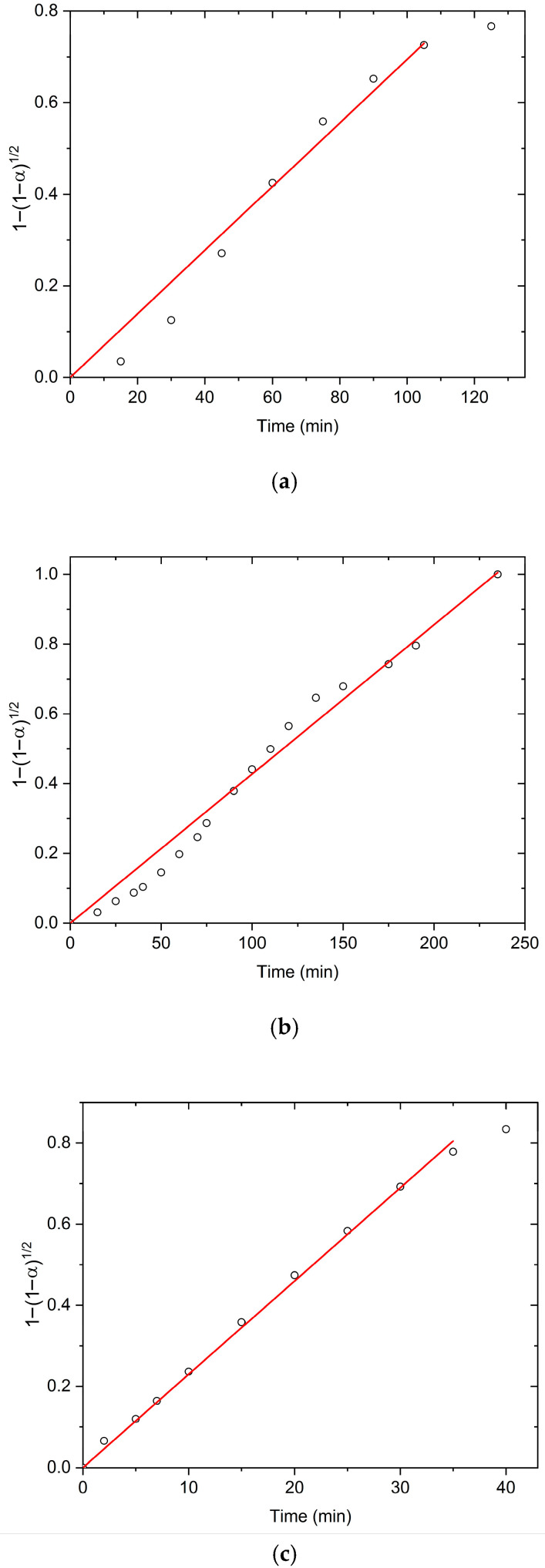
The isothermal dependence $1-{\left(1-\alpha \right)}^{\frac{1}{2}}$ with *t*, obtained under (**a**) TH, (**b**) US, and (**c**) MW field at 298 K.

In order to confirm the possibility of fitting experimental isothermal conversion kinetic swelling curves with the equation based on the boundary phase shrinkage model, the plots of $1-{\left(1-\alpha \right)}^{\frac{1}{2}}\;$ vs. time (Figure 5) for swelling in TH, US, and MW fields at 298 K are shown. The proposed mechanism is also most applicable during early and intermediate swelling stages. As the system approaches equilibrium, deviations from model predictions may appear, and its applicability becomes more limited. The suitability of the model near equilibrium must be evaluated case by case, as additional factors such as polymer relaxation and osmotic balance may dominate. In our case, the model remains valid up to approximately 80% of the equilibrium swelling degree, which represents a relatively wide applicability range.

The linearity of the isothermal dependence of $1-{\left(1-\alpha \right)}^{\frac{1}{2}}$ on time, at all investigated temperatures and applied external fields, across most α values, responsively confirms the validity of the shrinking boundary surface model to describe the swelling kinetics of PMAA hydrogel. Based on the obtained results, it may be proposed that the swelling kinetics of PMAA hydrogel in all applied fields is controlled by the rate of two-dimensional movement of the reactive polymer–water interface, which is formed during the interaction of the polymer network with water molecules [37]. Calculated swelling rate constants (*k*_2_) and correlation coefficients (R^2^) are summarized in Table 2.

The isothermal *k*_2_ values under US are lower than those under TH, while those under MW are notably higher. The increase in swelling temperatures leads to an increase in *k*_2_ values in all fields, following the Arrhenius equation. Activation energies (*E_a_*) and pre-exponential factors (ln *A*) calculated by applying the Peppas and boundary shrinkage models are shown in Table 3 for PMAA hydrogel swelling under TH, US, and MW.

The *E_a_* and ln *A* differ between models and fields. The order of magnitude is the same, but the Peppas model shows increased *E_a_* under US and MW vs. TH, while the boundary shrinkage model shows increased *E_a_* under MW but decreased under US. Although exact *E_a_* values are not the focus, these differences reinforce the idea that external fields induce structural changes in the PMAA network due to non-uniform absorption of ultrasonic and microwave energy.

Between the values of the swelling kinetic parameters for the boundary shrinkage model, a compensation effect correlating *E_a_* and ln *A* under different fields was found (R^2^ = 0.999), which is consistent with the Selective Energy Transfer (SET) model [38] that describes hydrogel swelling activation:

(5) $$ln\;A=-10.3+4.39\;\times 10^{-4}\;E_{a}\;\;\;(R^{2}\;=\;0.999)$$

The existence of this correlation implies a unique swelling activation mechanism across all applied fields driven by thermal activation and selective quantum transfer of discrete energy quanta (~2.5 kJ/mol) to the hydrogen bond oscillations (ν = 209 cm^−1^) between COOH groups in the PMAA network. Thus, hydrogel swelling requires proton transfer within H-bonds and segmental polymer chain motion.

The established changes in the kinetic parameters *E_a_* and ln *A* values for different fields reflect structural modifications of the xerogel/hydrogel network induced by each external field and altered water molecule mobility due to field effects.

## 4. Conclusions

This study demonstrates that both ultrasound and microwave fields significantly influence the equilibrium swelling degree (SD*_eq_*) and swelling kinetics of PMAA-based hydrogels. US produced the highest SD*_eq_* at constant temperature, while temperature increase enhanced SD*_eq_* under TH and MW conditions, but had minimal impact under US.

All swelling profiles were successfully described using the Peppas model, with field-dependent variations in kinetic parameters. The swelling rate constant (*k*) increased with temperature across all fields, reaching its maximum under the MW field. The highest activation energy (*E_a_*) was observed for MW, while US exhibited the highest pre-exponential factor (ln *A*).

The diffusion exponent (*n*) responded differently to temperature depending on the applied field, showing the highest values under US. Thermal activation occurred via selective energy transfer (SET) to specific polymer backbone vibrations, and the activation energy was found to be quantized.

Finally, a novel kinetic model incorporating boundary phase shrinking was applied, offering improved predictive accuracy in conditions where classical models fall short. A compensation effect was confirmed for this model.

These findings highlight the critical role of external fields in modulating hydrogel swelling and provide a solid foundation for tailoring material performance in applications such as drug delivery, sensors, and responsive systems. The introduced model enhances our ability to predict swelling behavior in complex environments, contributing to the broader understanding of non-equilibrium polymer dynamics.

## Figures and Tables

**Table 1:** Peppas kinetic parameters.

Field	Temp (K)	SD*_eq_* (g/g)	*k* (×10^−3^ min^−1^)	*n*
TH	298	443	1.44	1.44
	308	455	1.56	1.56
	313	459	2.03	1.56
US	298	413	1.54	1.26
	308	407	2.27	1.24
	313	403	3.84	1.15
MW	298	63.7	58.3	0.70
	308	83.5	71.7	0.76
	313	89.9	99.2	0.80

**Table 2:** Swelling rate constants (*k*_2_) and correlation coefficients (R^2^) under TH, US, and MW fields.

Field	Temp (K)	*k*_2_ (×10^−3^ min^−1^)	R^2^
TH	298	5.90	0.995
	308	6.95	0.990
	313	11.0	0.984
US	298	3.60	0.992
	308	4.28	0.992
	313	4.89	0.995
MW	298	13.8	0.999
	308	23.0	0.999
	313	39.6	0.999

**Table 3:** Kinetic parameters from Peppas (P) and boundary shrinkage (R2) models.

Field	Model	*E_a_* (kJ/mol)	ln *A* (s^−1^)
TH	P	15.96	−4.23
	R2	29.17	2.49
US	P	44.52	7.35
	R2	15.43	−3.50
MW	P	25.68	3.38
	R2	52.11	12.6

## Data Availability

The original contributions presented in this study are included in the article. Further inquiries can be directed to the corresponding author.

## Abbreviations

The following abbreviations are used in this manuscript:

DDS	Drug Delivery System
US	Ultrasound
MW	Microwave heating
PMAA	Poly(methacrylic acid)
TH	Thermal heating
SD	Swelling degree
ξ	Distance between the macromolecular chains
XRD	X-ray diffraction
SEM	Scanning electron microscopy
FTIR	Fourier-transform infrared spectroscopy
DMA	Dynamic mechanical analysis
SSNMR	Solid state nuclear magnetic resonance
SET	Selective energy transfer
